# The protective effect of URP20 on ocular *Staphylococcus aureus* and *Escherichia coli* infection in rats

**DOI:** 10.1186/s12886-022-02752-w

**Published:** 2022-12-30

**Authors:** Meng Li, Danli Xin, Jian Gao, Quanyong Yi, Jianshu Yuan, Yongbo Bao, Yan Gong

**Affiliations:** 1 School of Medicine, Ningbo University, Ningbo, 315042 China; 2 Department of Ophtalmology, Ningbo Eye Hospital, Ningbo, 315042 China; 3 College of Biological & Environmental Sciences, Zhejiang Wanli University, Ningbo, 315100 China; 4 Department of Ophtalmology, Medical College of Ningbo University, Ningbo Eye Hospital, No. 599, Beiming Cheng Road, Yinzhou District, Ningbo, 315042 China

**Keywords:** URP20, Infectious keratitis, Antimicrobial polypeptide, Ocular infection, Crassostrea *hongkongensis*

## Abstract

**Background:**

Infectious keratitis, a medical emergency with acute and rapid disease progression may lead to severe visual impairment and even blindness. Herein, an antimicrobial polypeptide from *Crassostrea hongkongensis*, named URP20, was evaluated for its therapeutic efficacy against keratitis caused by *Staphylococcus aureus (S. aureus)* and *Escherichia coli* (*E. coli)* infection in rats, respectively.

**Methods:**

A needle was used to scratch the surface of the eyeballs of rats and infect them with *S. aureus* and *E.coli* to construct a keratitis model. The two models were treated by giving 100 μL 100 μM URP20 drops. Positive drugs for *S. aureus* and *E. coli* infection were cefazolin eye drops and tobramycin eye drops, respectively. For the curative effect, the formation of blood vessels in the fundus was observed by a slit lamp (the third day). At the end of the experiment, the condition of the injured eye was photographed by cobalt blue light using 5 μL of 1% sodium fluorescein. The pathological damage to corneal tissues was assessed using hematoxylin–eosin staining, and the expression level of vascular endothelial growth factor (VEGF) was detected by immunohistochemistry.

**Results:**

URP20 alleviated the symptoms of corneal neovascularization as observed by slit lamp and cobalt blue lamp. The activity of *S. aureus* and *E.coli* is inhibited by URP20 to protect corneal epithelial cells and reduce corneal stromal bacterial invasion. It also prevented corneal thickening and inhibited neovascularization by reducing VEGF expression at the cornea.

**Conclusion:**

URP20 can effectively inhibit keratitis caused by *E.coli* as well as *S. aureus* in rats, as reflected by the inhibition of corneal neovascularization and the reduction in bacterial damage to the cornea.

**Supplementary Information:**

The online version contains supplementary material available at 10.1186/s12886-022-02752-w.

## Introduction

Bacterial keratitis is an acute or chronic corneal infection that may lead to catastrophic complications including corneal scarring, perforation of the eye, and worse, eventual loss of the entire eye and vision without being treated properly [1]. Bacterial keratitis accounts for approximately 90% of microbial keratitis [2, 3], whose aetiology and major contributing factors include contact lens wear, ocular trauma, ocular surface disease, long-term use of immunosuppressive drugs, and previous ophthalmic surgery [4], and *S. aureus* and *P. aeruginosa* are the main microorganisms causing bacterial keratitis [5]. Local injury or immune deficiency can lead to a wide diversity of pathogenic microorganisms, including bacteria, fungi, viruses, and protozoa infecting the cornea of the eye, all of which are associated with bacterial keratitis or corneal ulcers. Standard medical treatment for bacterial keratitis includes the use of topical or systemic antibiotics resulting in suboptimal visual acuity testing outcomes.

The standard clinical treatment pathway includes diagnosis of the underlying bacterial pathogen and therapy with appropriate antibiotics [4]. Broad-spectrum antibacterial drug therapy can be very beneficial in addressing multiple aetiologies and concomitant inflammation. However, pathogenic bacteria have reduced susceptibility and increased resistance to antibiotics due to the widespread use of broad-spectrum antibiotics [4]. This has become a challenge for health care systems worldwide [6–8]. Given the unfavourable outcomes of current treatments for infectious keratitis, novel therapeutic approaches are needed.

Typically, a variety of antimicrobial factors, especially in the tear film, protect the cornea from infections of the eye. The tear film is a mucus-containing fluid that covers the cornea and conjunctiva and contains many antimicrobial molecules produced by the innate and adaptive immune systems, such as antimicrobial peptides (AMPs) which have many important functions.

AMPs are mainly positively charged peptides containing no more than 100 amino acids and are produced by in microorganisms, animals and plants [9, 10]. Three important AMPs found in humans are defensins, tissue inhibitors, and cathelicidins [11]. In the human body, AMPs are produced by many different types of cells and are present in tissues and surfaces [12, 13]. Many endogenous AMPs are important in natural immunity for the defence against bacterial, fungal and viral pathogens [14]. Natural AMPs usually have broad-spectrum activity against gram-negative and gram-positive bacteria, fungi, eukaryotic parasites, and viruses [15]. Furthermore, a major advantage of AMPs is their ability to kill multidrug-resistant bacteria.

In addition to their antimicrobial effects, AMPs can modulate the inflammatory response and stimulate re-epithelialization during wound healing [16, 17]. The advantage of using AMPs for the treatment of ocular infections is that they can be applied directly to the infected area as a topical product [18]. Due to the tendency of bacteria to develop resistance to conventional antibiotics, new innovative eye drop formulations are urgently needed to effectively fight the bacteria causing ocular infections. AMPs, which are naturally possessed by humans, have an innate advantage for the treatment of ocular infections.

In our previous study, we isolated and identified an AMP from the plasma of oyster Hong Kong, URP20 peptide with powerful antibacterial and antifungal effects. URP20 demonstrated a significant ability to damage cell membranes of both gram-negative and Gram-positive bacteria and fungi, indicating a wide range of antimicrobial activity against microorganisms. URP20 was not cytotoxic or pro-inflammatory to mammalian cells and mice in the bactericidal concentration range, further supporting its safe use as a naturally occurring antimicrobial agent [19].

The purpose of this study was to investigate the antimicrobial efficacy and ocular safety considerations of the URP20. The toxicity of the peptide to rat corneal epithelial cells (HCEC) was evaluated and compared with the antibacterial effect of commonly used eye drops in the clinic.

## Materials and methods

### Experimental materials

The antibacterial peptide URP20 was retrieved from the marine mollusc *Crassostrea hongkongensis plasma* peptide library [19], constructed by the group and synthesized by Hefei National Peptide Biotechnology Co., Ltd. URP20 has a molecular weight size of 1.41 kDa and an amino acid sequence of MDAIKKKMLAMK, and is dissolved directly in sterile PBS when used. Giemsa stain solution was purchased from Solarbio and VEGF antibody was purchased from ABclonal (Cat. No. A12303).

### Establishment of an ocular bacterial infection model in rats

Sixty 250–300 g clean-grade Sprague–Dawley (SD) male rats were purchased from Shanghai JieSiJie Laboratory Animal Co., Ltd. The animals were housed in a specific pathogen free-grade animal house for one week of acclimatization feeding, during which they were fed with water ad libitum and kept under a 12-h light/dark environment. The eyes of all rats were examined to ensure that there were no defects by slit lamp examination prior to model establishment. The rats were randomly divided into model control groups (Groups A1 and A2), antibiotic-treated groups (Groups B1 and B2), and URP20-treated groups (Groups C1 and C2), with 10 rats in each group. *S. aureus* and *E. coli* bacteria were cultured in LB medium at 37 °C until the exponential growth period and the inoculum was adjusted according to the OD 650 nm value to achieve approximately 10^7^ colony units (CFUs).

The rats were all anaesthetized by intraperitoneal injection of 2% sodium phenobarbital (35 mg/kg), and the corneal epithelium of the left eye was scraped using a 26-gauge needle to form a superficial wound without destroying the stromal layer. In the A1, B1, and C1 groups, 20 μL of an equal concentration of *S. aureus* suspension was applied to the corneal surface for immediate infection, while for Groups A2, B2, and C2, 20 μL of an equal concentration of *E.coli* suspension was used instead of the *S. aureus* suspension. After immediate infection in A1 and A2 as model control groups, plexiglass sheets were used to cover the surface of the eye to prevent loss of bacterial fluid, and the upper and lower eyelids were sutured until the sutures were removed 24 h later, and saline drops were given every 2 h 6 times a day for 2 days. Group B1: After the immediate infection, the surface of the eye was covered with a plexiglass sheet and an equal volume of cefazolin drops was added and the upper and lower eyelids were kept sutured for 24 h. Cefazolin drops were given once every 2 h 6 times a day for 2 days. Group B2: All procedures were the same as those for group B1 except that tobramycin (TOB) eye drops were replaced with cefazolin eye drops. Groups C1 and C2: The treatment was performed with 100 μL URP20 concentration of 100 μM as eye drops, and the other steps were the same as those of group B1 and B2. At the end of the experiment, the rats were killed by injection of 60 mg/kg sodium phenobarbital for deep anaesthesia. The serum was obtained by centrifuging the blood collection from the main abdominal vein of rats, and stored at -80 °C. The left eyes from the experimental groups and the right eyes (normal controls) were fixed and used to make paraffin sections. Some corneal tissues were snap-frozen in liquid nitrogen and stored at -80 °C to prepare for assays such as western blotting and qPCR.

All procedures involving animals were performed in strict accordance with the statement on the Use of Animals in Ophthalmic and Vision Research and the recommendations in the National Institutes of Health Guide for the Care and Use of Laboratory Animals. The protocol was approved by the Laboratory Animal Protection and Use Committee of Ningbo University and conforms to the guidelines of the Animal Research: Reporting of in vivo Experiments (ARRIVE) for the use of laboratory animals.

### Slit lamp microscope examination

The rats were anaesthetized before the experiment and 72 h after administration, and the changes in ocular neovascularization and ocular turbidity were observed with a slit lamp. The rats were all anaesthetized by intraperitoneal injection of 2% sodium phenobarbital (35 mg/kg). The ratio of neovascularization area to total corneal area was calculated from the corneal edge. The results were calculated by IPP 6.0 image processing and analysis software, and each sample was tested three times.

### Corneal fluorescein sodium staining

To evaluate the degree of bacterial infection, staining was performed after anaesthesia for 3 min by adding 5 μL of 1% sodium fluorescein (Solarbio) drop by drop to the corneal area of the eye in rats. The eyeball status of the rats was then photographed by cobalt blue light. The results were calculated by IPP 6.0 image processing and analysis software, and each sample was tested three times.

### The viable count of bacteria

To determine the antibacterial effect, rats were anaesthetized on Day 3, and 10 μL of sterile PBS was added into the surface of the eye for the experimental group in rats before aspirating the mixture of PBS and eye secretions (10 μL in total) with a gun tip. The 10 μL mixed droplets were then coated into LB solid medium and incubated at 37 °C overnight before photography. The number of plate colonies formed was counted by IPP 6.0 software, and each sample was tested three times.

### Giemsa staining

To assess the protective effect of AMPs on the cells of the ocular surface, collected teardrops collected were added to slides to make smears, them allowing to dry naturally before 100 μL of Giemsa staining solution was added dropwise for 30 s. Then, another 200 μL of PBS was added and rinsed with tap water after 5 min. The results were photographed by light microscopy (DM500, Leica).

### HE staining

Freshly taken ocular tissues were fixed in 10% neutral formaldehyde solution for 24 h and dehydrated in alcohol. After paraffin embedding, 5-μm paraffin sections were made. Animal tissue sections were subjected to paraffin melting in an oven at 65 °C for 20 min. Tissue sections were immersed in xylene for 20 min before graded alcohol rehydration, then followed by heamatoxylin staining for 3 min followed by tap water rinsing for 1 min. Differentiation of staining was performed using 1% alcohol hydrochloric acid for 3 s. Before eosin staining for 5 s, tissues were stained blue with 0.1% NaHCO_3_. The sections were dried after dehydration in 95% alcohol and sealed with neutral gum after xylene transparency. Photographs were taken by light microscopy, and each sample was tested three times.

### Immunohistochemistry

Paraffin sections were dewaxed and rehydrated, and placed in 0.01 M citrate buffer (pH = 6.0). Antigen repair was performed in an autoclave at 121 °C for 20 min. After recovery to room temperature, endogenous peroxidase was inactivated by dropwise addition of 3% H_2_O_2_ for 15 min and antigen was blocked with 1% BSA (dissolved in PBS) for 30 min. The primary antibody rabbit anti-rat VEGF (1:50 dilution, dissolved in 1% BSA) was added and incubated overnight at 4 °C. Secondary antibody coupled to horseradish peroxidase-conjugated AffiniPure goat anti-rabbit IgG (1:100 dilution) was added dropwise and incubated for 60 min at room temperature. DAB chromogenic solution was used for colour development. Hematoxylin restaining followed by alcohol gradient dehydration. The slices were sealed with neutral gum after clearing in xylene. The immunohistochemical results were observed by light microscopy and analysed using IPP 6.0 softwares, and each sample was tested three times.

### Statistical methods

All experimental data are presented as the arithmetic mean of 10 measurements ± standard deviation, and the related analysis and statistics were performed by GraphPad Prism 8.0. Comparisons between two groups were analysed by *t*-*test*. * represents *P* < 0.05, # represents *P* < 0.05, and the difference is statistically significant.

## Results

### Comparison of the antibacterial effect of URP20 on ocular infection in rats

**Fig. 1:**
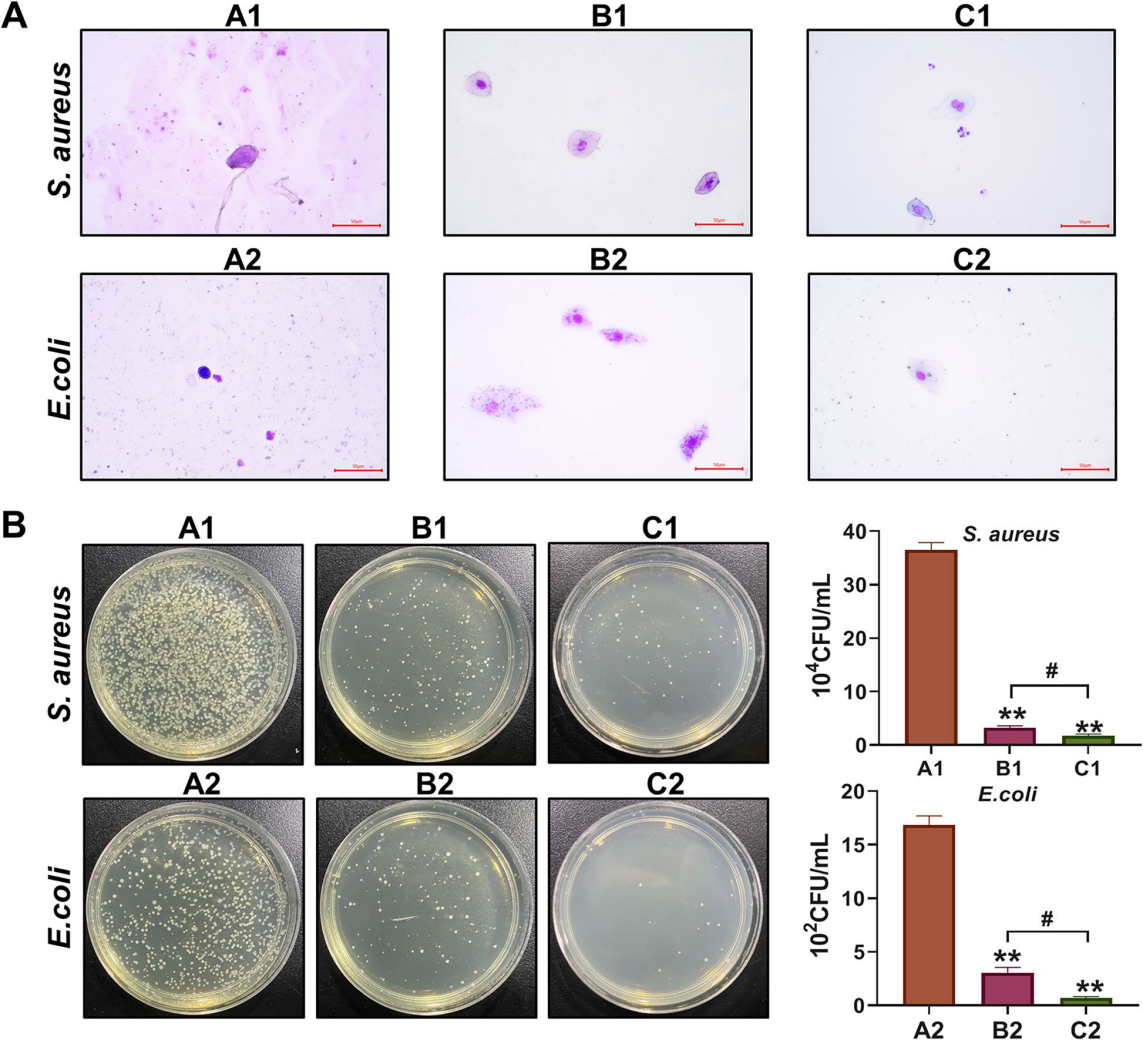
Analysis of cellular status and bacterial content in rat eyes. **A** Staining of secretions in rat tear fluid by Giemsa. **B** Detection of bacterial count levels on rat ocular surface by spread plate culture. A1: *S. aureus* model group, B1: *S. aureus* + cefazolin group, C1: *S. aureus* + URP20 group, A2: E. coli model group, B2: *E. coli* + TOB group, C2: *E. coli* + URP20 group. **P < 0.01, compared with A1 or A2 group. #*P* < 0.05, there is a statistical difference between the two groups

Figure 1A shows Giemsa staining of cells in rat eye secretions. The results showed that the number of normal ocular epithelial cells in the A1 *S. aureus* model group was reduced, the nuclei were lost and the cell morphology was pyknotic. After cefazolin eye drop treatment in the B1 group, the number of normal epithelial cells increased, and a small number of cells invaded the cell interior. After the treatment with URP20 in the C1 group, the number of normal epithelial cells increased, and no obvious bacterial invasion was seen in the cell interior, but some epithelial cells presented nuclear atypia. In the A2 *E. coli* model group, the epithelial cells exhibited pyknotic and nuclear atypia. In the B2 TOB-treated group, the number of epithelial cells recovered, but there was a large number of bacterial invasions in the cells. No bacterial invasion was observed in the normal cells in the C2 group which was less than B2. Figure 1B shows the colony formation of the coated plate, which shows that the number of colonies formed was the lowest in the C1 and C2 URP20-treated groups.

### Protective effect of URP20 in the prevention and treatment of bacterial infection after eye injury

**Fig. 2:**
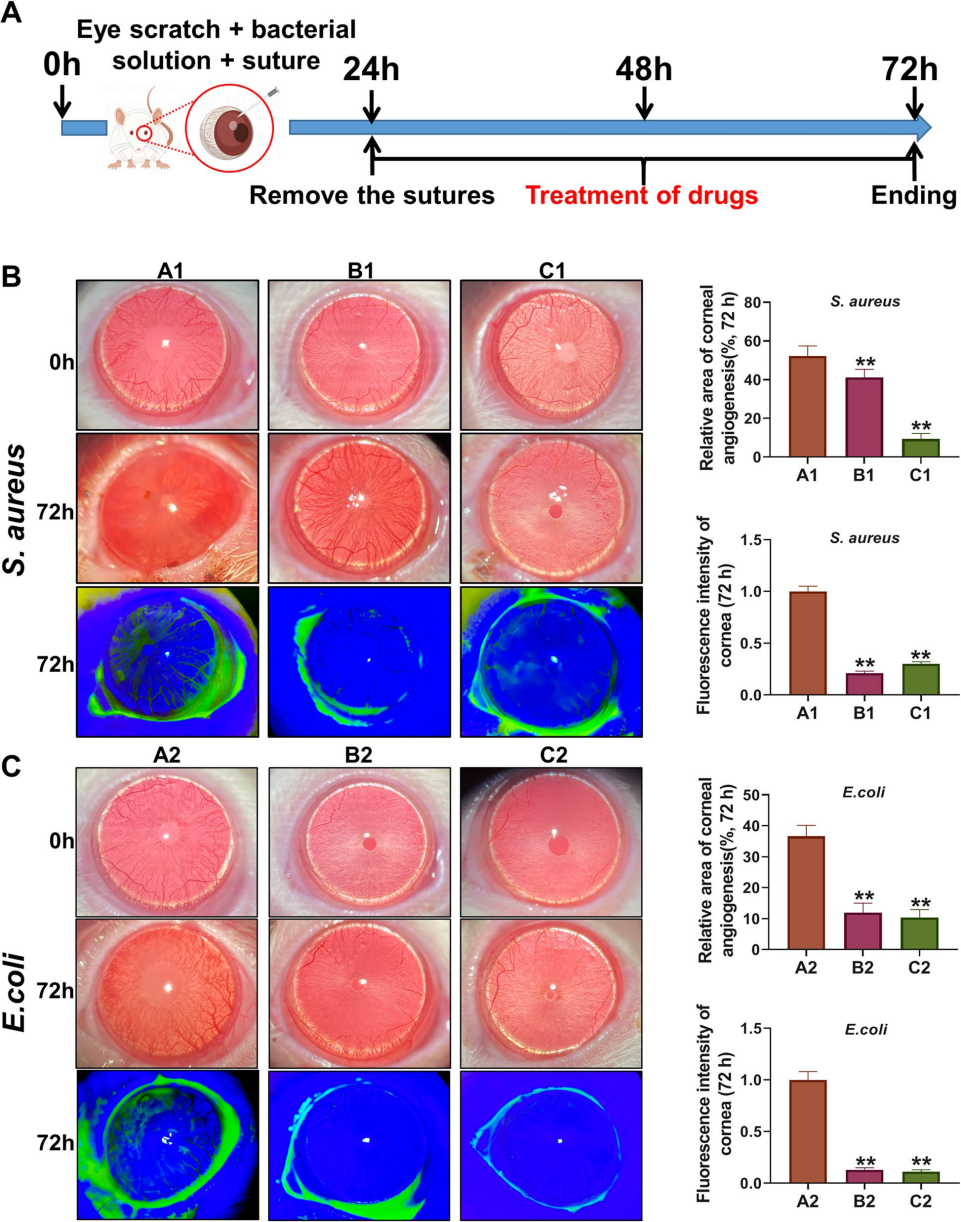
Slit-lamp photographing of eyes of rats. **A** Flow chart of animal experiments. **B** Photographs of *S. aureus* infection in rats. **C** Photographs of *E. coli* infection in rats. The blue background was taken with a cobalt blue slit lamp. **P < 0.01, compared with A1 or A2 group

**Fig. 3:**
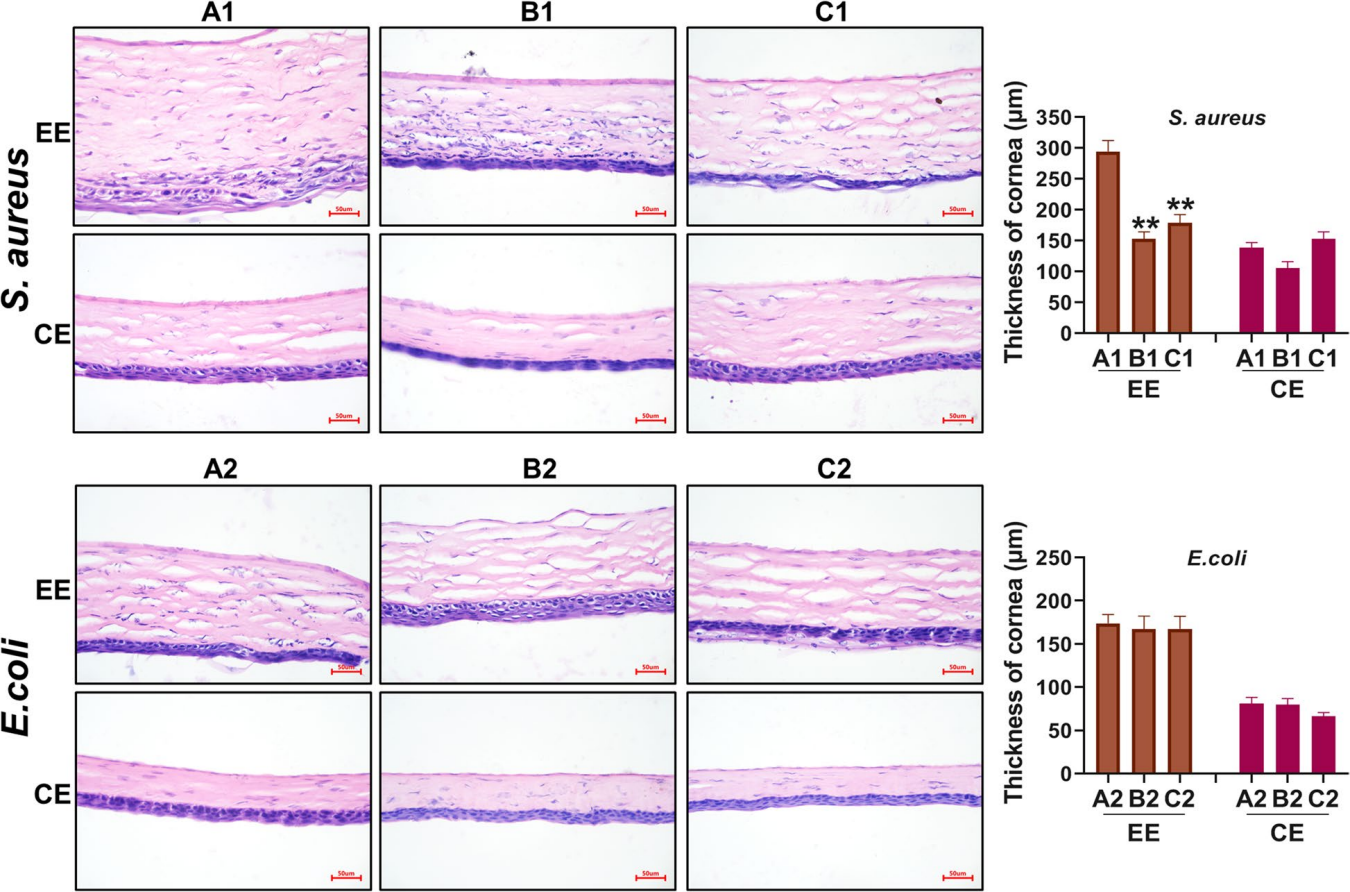
The results of the pathological examination by HE staining. EE: experimental eyeball (left eye); CE: normal control eyeball (right eye). **P < 0.01, compared with the A1 group

Figure 2A shows the flow chart of the whole animal experiment. As shown in Fig. 2B-C, the rat eyeballs in Groups A1 and A2 showed turbidity and vascular proliferation after 72 h of infection with *S. aureus* and *E. coli*, respectively. Large areas of bacterial invasion damage (green fluorescent areas on the surface of the eye) could be seen under cobalt blue light. The area of sodium fluorescein staining was reduced in both the C1 and C2 groups after treatment with URP20, and the effect was even stronger in *E. coli*. The results of the pathological examination by HE staining (Fig. 3) showed that the membrane structure displayed by the rat eyes was significantly thickened after the infection with *S. aureus* and *E. coli*, especially in the *S. aureus* model group. The A1 *S. aureus* model group showed massive cell death on the surface of the eyes and increased membrane interstitium, which was consistent with the characteristics of cloudy eyes under a slit lamp. In the C1 group, the corneal structure was significantly thinner than that of the A1 group, and there were a few dead cells in the membrane mesenchymal. The cellular structure of the surface of the eyes in Group C2 was more intact, and no significant bacterial invasion was seen in the mesenchymal membrane.

### Effect of URP20 on VEGF expression levels after ocular bacterial infection

**Fig. 4:**
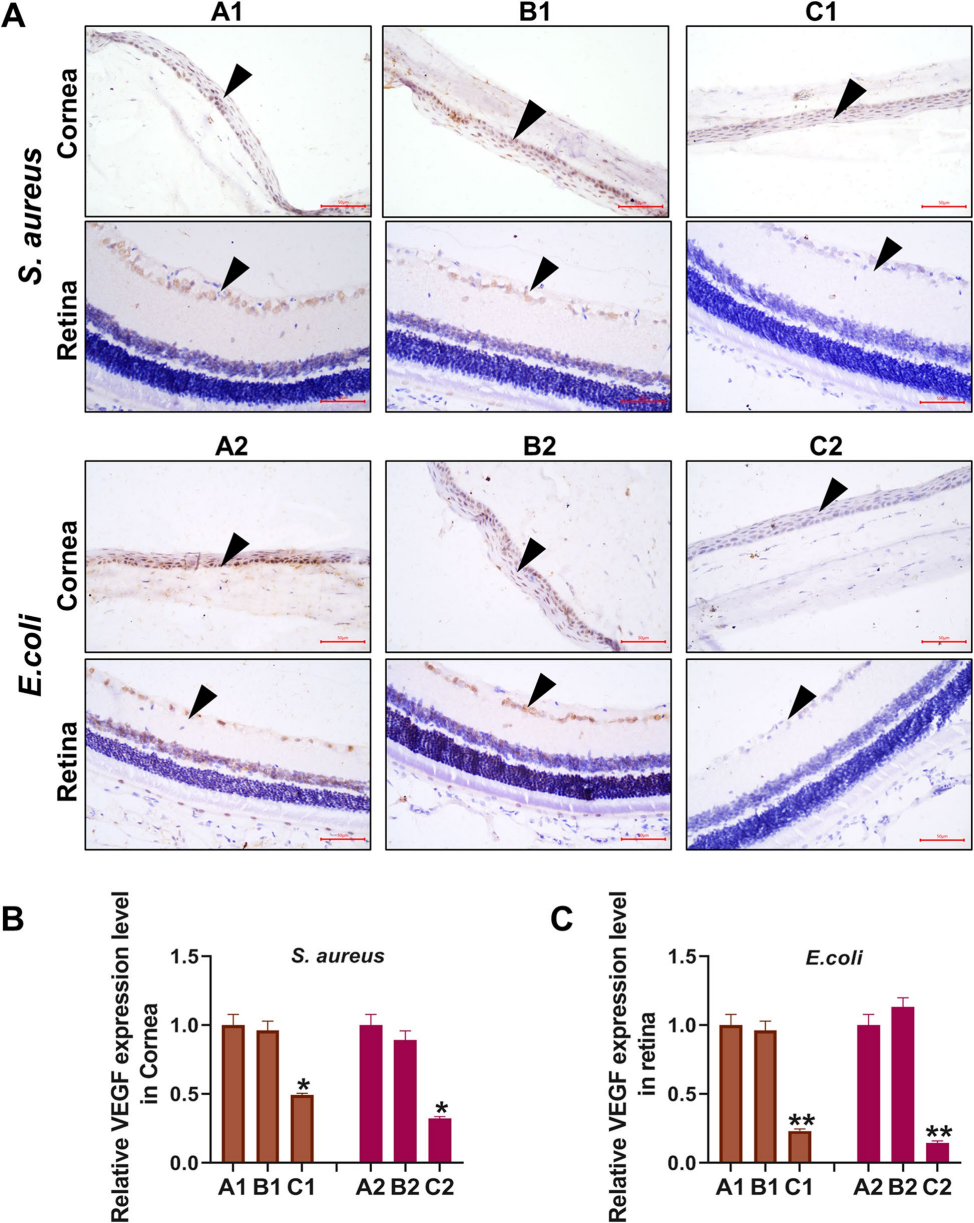
Ocular VEGF was detected by immunohistochemistry. **A** The VEGF expression levels on the infected eyeball in rats shown by immunohistochemy (The brown part indicated by the black arrow). **B** Detection and localization of VEGF expression levels in the cornea by IPP6.0. **C** Detection and localization of VEGF expression levels in the retina by IIPP6.0. *P < 0.05, **P < 0.01, compared with A1 or A2 group

Figure 4 shows the immunohistochemical detection of VEGF expression levels on the surface and fundus of the eyeball, and the results suggest that the treatment with URP20 significantly reduced the expression levels of VEGF on the surface and fundus of the eye, and the differences were statistically significant compared with A1 and A2 (*P* < 0.05).

## Discussion

AMPs have been among the compounds that have been shown to have antibiotic properties in recent years. These biomolecules can destroy pathogens by stimulating the host's innate immunity [20]. The advantages of AMPs over conventional antibiotics include a lower rate of induced resistance, a wider range of pathogen effects, specificity for Gram-positive or Gram-negative bacteria, less host toxicity, synergistic antimicrobial effects with other antibiotics, and the ability to rapidly exert their bactericidal effects [21]. Therefore, increasing numbers of researchers are paying attention to them. URP20, an antimicrobial peptide identified and identified by our team members in previous studies, has a strong anti-*E.coli* and *S.ureus* effect. Therefore, URP20 will become the focus of our follow-up research on the treatment of ophthalmic infectious diseases [19].

*S. aureus* is one of the most important pathogenic bacteria of keratitis, with fibronectin-binding protein that enables bacteria to adhere and invade epithelial cells [22]. The mucin layer and tight intercellular junctions of the corneal epithelium are the main barriers that prevent *S. aureus* from binding and penetrating the cornea. Disruption of these barriers can significantly increase susceptibility to *S. aureus* infection and lead to *S. aureus* keratitis [23, 24]. *S. aureus* has developed into difficult to treat resistant strains [25, 26], that are responsible for 5–36% of corneal ulcers [27]. In addition, gram-negative bacteria are also important contributors to ocular infections. Among the gram-negative bacteria, *E. coli* and Enterobacter spp. are commonly isolated from conjunctivitis, dacryocystitis, and keratitis [28, 29]. In addition, another study from Egypt reported the isolation of *E. coli* and *A. lwoifi* (*Acinetobacter lwoifi*) from cases of chronic dacryocystitis [30].

The construction of the ocular bacterial infection model was improved on the basis of Tanweer et al. [31]. After the corneal injury in SD rats, *S. aureus* and *E. coli* were inoculated, and the upper and lower eyelids were sutured with glass sheets covered with radian to prevent loss of the bacterial fluid from losing. The reason for the improvement was that no obvious bacterial ocular infection was found in the model based on Tanweer's research method, which may also be caused by the differences in models between rats and mice due to their own immune systems and environmental factors. Encouragingly, after URP20 treatment, the ocular infection in rats recovered significantly, with less ocular neovascularization and no drainage or other reactions. Pathologically, URP20 effectively maintained the structure and morphology of corneal epithelial cells while reducing the bacterial invasion of the corneal stroma layer.

To further explore the antibacterial effect and cytotoxicity of URP20 on eyes, we combined Giemsa staining and plate colony formation experiments. The results showed that 100 μM of URP20 showed an inhibitory effect on *S. aureus* and *E. coli* that was even stronger than that of the eye drops cefazolin and TOB. This may be related to the property of URP20, where high concentrations of URP20 take on a gel-like appearance at 37 °C, and it is this property that may allow URP20 to linger longer and exert a longer-lasting bacteriostatic effect when dropped into the rat eye. URP20 treatment facilitated the recovery of corneal epithelial tissue.

The cornea is the external barrier of the eye and is transparent and avascular under healthy conditions. However, an imbalance between angiogenic and antiangiogenic stimuli following severe injury or chronic inflammation can lead to abnormal amounts of proangiogenic factors. For example, in the case of excess vascular endothelial growth factor (VEGF), a normally avascular cornea may become vascularized [32]. In addition, overexpression of VEGF indirectly induces lymphangiogenesis, whereas corneal lymphangiogenesis cannot disturb visual acuity [33, 34]. As blood and lymphatic vessels can enter the cornea from adjacent vascularized tissue, this leads to vision loss and passive immune response [35]. Neovascularization is a common complication of corneal infection, and corneal angiogenesis is a common endpoint in different ocular surface diseases, including infected corneas. Although corneal angiogenesis is beneficial in preventing stromal melting, promoting wound healing and eliminating infection, it brings about persistent inflammation, oedema, lipid deposition and tissue scarring. This comes at the cost of reducing corneal transparency, which can lead to poor vision [36]. Therefore, in bacterial infectious keratitis, the level of corneal neovascularization is an important therapeutic consideration in addition to bacterial suppression [37].

Corneal opacity and neovascularization were found after *S. aureus* infection, as reported by Cicih and Nicole [38] et al., which is also the case in this study. As a pathogen of eye infection, *E.coli* is rarely reported. In this study, no obvious corneal turbidity was found, but it caused significant neovascularization. After treatment with URP20, the symptoms of corneal neovascularization caused by *S. aureus* and *E. coli* were significantly relieved. After URP20 treatment, the level of VEGF protein secreted by the corneal stromal layer and corneal epithelial cells decreased, as shown by immunohistochemy. The advantage of this experiment is that URP20 is a polypeptide from food with higher safety and can be used in human experiments. But there are also some limitations. This experiment mainly studies the antibacterial effect, and whether it has an impact on inflammatory factors remains to be studied.

In this study, the effective antibacterial effect and corneal epithelial cytoprotective function of URP20 in ocular infections were verified in an ocular *S. aureus* and *E. coli*-infected rat model. It was also found that URP20 could inhibit the expression of VEGF in the cornea to suppress corneal neovascularization. In addition, the URP20 at high concentrations can form a gel in vivo, which enhances its duration of action in the eye. Therefore, we believe that URP20 could be a candidate for the treatment of ocular infections and deserves further examination. We will further explore the safe applicable dose for the use of URP20 subsequently for better application development at a later stage.

## Supplementary Information

**Additional file 1. **The inhibition of URP20 with different concentrations on five different bacteria.

## Data Availability

All data generated or used during this study are included in this published article, still, further details are available when requested from the corresponding author.

## References

[1] Austin A, Lietman T, Rose-Nussbaumer J (2017). Update on the Management of Infectious Keratitis. Ophthalmology.

[2] Lakhundi S, Siddiqui R, Khan NA (2017). Pathogenesis of microbial keratitis. Microb Pathog.

[3] Mun Y, Kim MK, Oh JY (2019). Ten-year analysis of microbiological profile and antibiotic sensitivity for bacterial keratitis in Korea. PLoS ONE.

[4] Khor WB, Prajna VN, Garg P, Mehta JS, Xie L, Liu Z (2018). The asia cornea society infectious keratitis study: a prospective multicenter study of infectious keratitis in asia. Am J Ophthalmol.

[5] Chojnacki M, Philbrick A, Wucher B, Reed JN, Tomaras A, Dunman PM (2019). Development of a broad-spectrum antimicrobial combination for the treatment of staphylococcus aureus and pseudomonas aeruginosa corneal infections. Antimicrob Agents Chemother..

[6] Zhang W, Wu Y, Liu L, Xiao X, Cong Z, Shao N (2021). The membrane-targeting mechanism of host defense peptides inspiring the design of polypeptide-conjugated gold nanoparticles exhibiting effective antibacterial activity against methicillin-resistant Staphylococcus aureus. J Mater Chem B.

[7] Lalitha P, Manoharan G, Karpagam R, Prajna NV, Srinivasan M, Mascarenhas J (2017). Trends in antibiotic resistance in bacterial keratitis isolates from South India. Br J Ophthalmol.

[8] Cabrera-Aguas M, Khoo P, George C, Lahra MM, Watson SL (2020). Antimicrobial resistance trends in bacterial keratitis over 5 years in Sydney. Australia Clin Exp Ophthalmol.

[9] Mahlapuu M, Håkansson J, Ringstad L, Björn C (2016). Antimicrobial peptides: an emerging category of therapeutic agents. Front Cell Infect Microbiol.

[10] Wang G, Mishra B, Lau K, Lushnikova T, Golla R, Wang X (2015). Antimicrobial peptides in 2014. Pharmaceuticals (Basel).

[11] Ong PY, Ohtake T, Brandt C, Strickland I, Boguniewicz M, Ganz T (2002). Endogenous antimicrobial peptides and skin infections in atopic dermatitis. N Engl J Med.

[12] Epand RM, Vogel HJ (1999). Diversity of antimicrobial peptides and their mechanisms of action. Biochim Biophys Acta.

[13] Wang G (2014). Human antimicrobial peptides and proteins. Pharmaceuticals (Basel).

[14] Zasloff M (2002). Antimicrobial peptides of multicellular organisms. Nature.

[15] Hancock RE (2001). Cationic peptides: effectors in innate immunity and novel antimicrobials. Lancet Infect Dis.

[16] Hilchie AL, Wuerth K, Hancock RE (2013). Immune modulation by multifaceted cationic host defense (antimicrobial) peptides. Nat Chem Biol.

[17] Cederlund A, Gudmundsson GH, Agerberth B (2011). Antimicrobial peptides important in innate immunity. FEBS J.

[18] Gordon YJ, Romanowski EG, McDermott AM (2005). A review of antimicrobial peptides and their therapeutic potential as anti-infective drugs. Curr Eye Res.

[19] Mao F, Bao Y, Wong NK, Huang M, Liu K, Zhang X (2021). Large-scale plasma peptidomic profiling reveals a novel, nontoxic, crassostrea hongkongensis-derived antimicrobial peptide against foodborne pathogens. Mar Drugs..

[20] Zhang QY, Yan ZB, Meng YM, Hong XY, Shao G, Ma JJ (2021). Antimicrobial peptides: mechanism of action, activity and clinical potential. Mil Med Res.

[21] Seyfi R, Kahaki FA, Ebrahimi T, Montazersaheb S, Eyvazi S, Babaeipour V (2020). Antimicrobial Peptides (AMPs): roles, functions and mechanism of action. Int J Pept Res Ther.

[22] Jett BD, Gilmore MS (2002). Internalization of Staphylococcus aureus by human corneal epithelial cells: role of bacterial fibronectin-binding protein and host cell factors. Infect Immun.

[23] Zhang Z, Abdel-Razek O, Hawgood S, Wang G (2015). Protective role of surfactant protein D in ocular staphylococcus aureus infection. PLoS ONE.

[24] Ricciuto J, Heimer SR, Gilmore MS, Argüeso P (2008). Cell surface O-glycans limit Staphylococcus aureus adherence to corneal epithelial cells. Infect Immun.

[25] Chang VS, Dhaliwal DK, Raju L, Kowalski RP (2015). Antibiotic resistance in the treatment of staphylococcus aureus Keratitis: a 20-Year Review. Cornea.

[26] Ung L, Bispo P, Shanbhag SS, Gilmore MS, Chodosh J (2019). The persistent dilemma of microbial keratitis: Global burden, diagnosis, and antimicrobial resistance. Surv Ophthalmol.

[27] Ting D, Ho CS, Deshmukh R, Said DG, Dua HS (2021). Infectious keratitis: an update on epidemiology, causative microorganisms, risk factors, and antimicrobial resistance. Eye (Lond).

[28] Teweldemedhin M, Gebreyesus H, Atsbaha AH, Asgedom SW, Saravanan M (2017). Bacterial profile of ocular infections: a systematic review. BMC Ophthalmol.

[29] Hemavathi, Sarmah P, Shenoy P (2014). Profile of microbial isolates in ophthalmic infections and antibiotic susceptibility of the bacterial isolates: a study in an eye care hospital, bangalore. J Clin Diagn Res..

[30] Amin RM, Hussein FA, Idriss HF, Hanafy NF, Abdallah DM (2013). Pathological, immunohistochemical and microbiologicalal analysis of lacrimal sac biopsies in patients with chronic dacrocystitis. Int J Ophthalmol.

[31] Zaidi T, Zaidi T, Yoong P, Pier GB (2013). Staphylococcus aureus corneal infections: effect of the Panton-Valentine leukocidin (PVL) and antibody to PVL on virulence and pathology. Invest Ophthalmol Vis Sci.

[32] Nicholas MP, Mysore N (2021). Corneal neovascularization. Exp Eye Res.

[33] Asano D, Hokazono M, Hirano S, Morita A, Nakahara T (2019). Cellular mechanisms of angiogenesis in neonatal rat models of retinal neurodegeneration. Int J Mol Sci..

[34] Nakao S, Hafezi-Moghadam A, Ishibashi T (2012). Lymphatics and lymphangiogenesis in the eye. J Ophthalmol.

[35] Hadrian K, Willenborg S, Bock F, Cursiefen C, Eming SA, Hos D (2021). Macrophage-mediated tissue vascularization: similarities and differences between cornea and skin. Front Immunol.

[36] Feizi S, Azari AA, Safapour S (2017). Therapeutic approaches for corneal neovascularization. Eye Vis (Lond).

[37] Trikha S, Parikh S, Osmond C, Anderson DF, Hossain PN (2014). Long-term outcomes of Fine Needle Diathermy for established corneal neovascularisation. Br J Ophthalmol.

[38] Broekema NM, Larsen IV, Naruzawa ES, Filutowicz M, Kolb AW, Teixeira LB, et al. A Mouse Model of Multi-Drug Resistant Staphylococcus aureus-induced Ocular Disease. J Ocul Biol. 2016;4(2):1-5. 10.13188/2334-2838.1000026. 10.13188/2334-2838.1000026 PMC5123590 27896297

