# Supplementary material for: The protective effect of URP20 on ocular Staphylococcus aureus and Escherichia coli infection in rats
Source: BMC Ophthalmol. 2022 Dec 30;22:517. doi: 10.1186/s12886-022-02752-w (PMC9801630; doi:10.1186/s12886-022-02752-w)
Supplement: Supplementary file 1 — Additional file 1. The inhibition of URP20 with different concentrations on five different bacteria. [file 12886_2022_2752_MOESM1_ESM.doc]

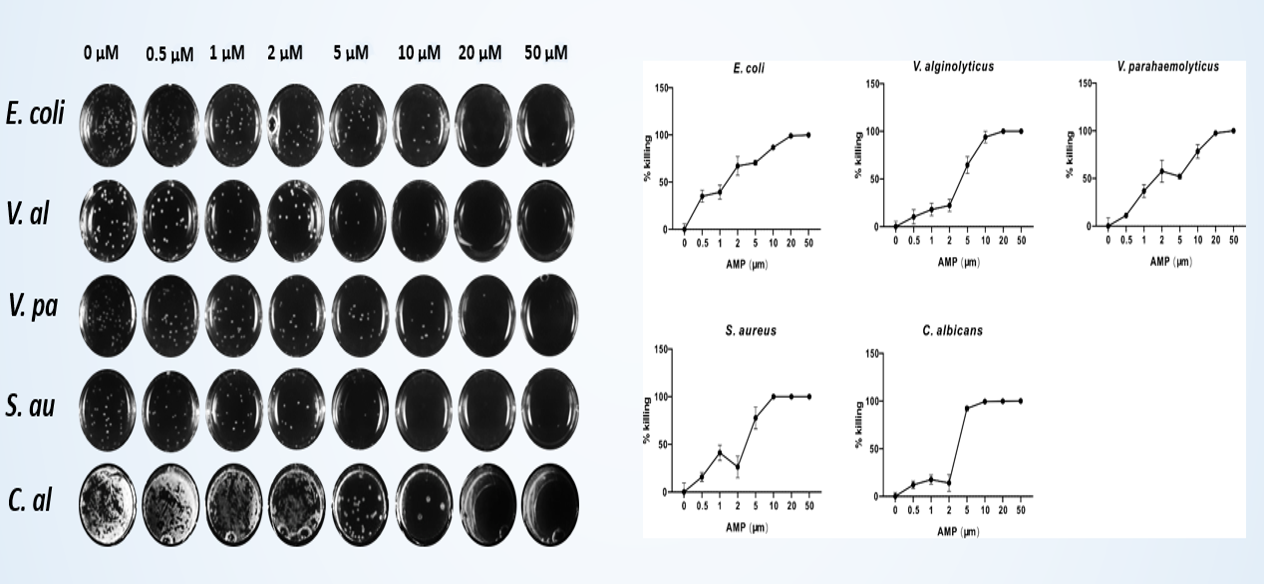


Supplementary material: The inhibition of URP20 with different concentrations on five different bacteria.
